# Compliment Graphene Oxide Coating on Silk Fiber Surface via Electrostatic Force for Capacitive Humidity Sensor Applications

**DOI:** 10.3390/s17020407

**Published:** 2017-02-19

**Authors:** Kook In Han, Seungdu Kim, In Gyu Lee, Jong Pil Kim, Jung-Ha Kim, Suck Won Hong, Byung Jin Cho, Wan Sik Hwang

**Affiliations:** 1 Department of Materials Engineering, Korea Aerospace University, Goyang 10540, Korea; kooookin@gmail.com (K.I.H.); seungdukim@gmail.com (S.K.); leeig@kau.ac.kr (I.G.L.); 2 Division of High Technology Materials Research & Molecular Materials Research Team, Korea Basic Science Institute, Busan 168-230, Korea; jpkim@kbsi.re.kr (J.P.K.); jhkim08@kbsi.re.kr (J.-H.K.); 3 Department of Cogno-Mechatronics Engineering, Department of Optics and Mechatronics Engineering, College of Nanoscience and Nanotechnology, Pusan National University, Busan 46241, Korea; swhong@pusan.ac.kr; 4 Department of Electrical Engineering, KAIST, Daejeon 34141, Korea; bjcho@kaist.edu

**Keywords:** graphene oxide coating, electrostatic force, capacitive sensor, humidity sensor

## Abstract

Cylindrical silk fiber (SF) was coated with Graphene oxide (GO) for capacitive humidity sensor applications. Negatively charged GO in the solution was attracted to the positively charged SF surface via electrostatic force without any help from adhesive intermediates. The magnitude of the positively charged SF surface was controlled through the static electricity charges created on the SF surface. The GO coating ability on the SF improved as the SF’s positive charge increased. The GO-coated SFs at various conditions were characterized using an optical microscope, scanning electron microscopy (SEM), energy-dispersive X-ray spectroscopy (EDS), Raman spectroscopy, and LCR meter. Unlike the intact SF, the GO-coated SF showed clear response-recovery behavior and well-behaved repeatability when it was exposed to 20% relative humidity (RH) and 90% RH alternatively in a capacitive mode. This approach allows humidity sensors to take advantage of GO’s excellent sensing properties and SF’s flexibility, expediting the production of flexible, low power consumption devices at relatively low costs.

## 1. Introduction

The onset of the internet of things (IoT) and virtual reality (VR) demands the development of flexible, portable devices [1,2]. These devices will require flexible, low power consumption, low cost humidity sensors [3,4,5]. Thus far, various materials, including polymers [6,7,8], metal oxides [9,10,11], porous materials [12,13,14], and nano-materials [15,16], have been investigated for advanced humidity sensors. Unlike conventional rigid materials, flexible detection objects have the potential to accelerate the realization of flexible electronics and open a new development path for various humidity sensors for IoT and VR applications [17,18]. Recently, two-dimensional (2D) materials such as graphene and graphene oxide (GO) have been studied intensively due to their extraordinary flexibility and high surface-area-to-volume ratios [19,20,21,22,23,24,25,26,27]. In particular, GO has an advantage over graphene. Whereas graphene has hydrophobic properties, GO has hydrophilic properties, making it beneficial for detecting polar gases like water molecules [28,29]. Furthermore, GO can be combined with flexible materials to create highly flexible hybrid structures with excellent gas sensing properties.

In fact, several approaches have been investigated for this purpose via implementing adhesive intermediates between GO and selected objects for better adhesion [30,31,32] Meanwhile, power consumption during both operational and standby conditions is another important concern since advanced humidity sensors are often adapted for mobile devices whose functions are highly limited with respect to power consumption and battery capacity [33]. As such, there is a need for low power consumption sensing mechanisms that can be demonstrated via capacitive mode. Compared to conventional conductivity or resistivity sensing, capacitive sensing can dramatically reduce power consumption during standby and operation modes [34]. In addition, it was reported that the capacitive type is less affected by the temperature variation than the resistive type [35]. Finally, this method is cost-effective since GO can be easily and cheaply mass-produced, and silk fiber (SF) can be coated with GO without any help from adhesive intermediates [36]. In this work, GO-coated SF is presented for flexible and wearable applications unlike conventional solid detection materials. The quality and quantity of the GO were controlled by varying the electrification force on the SF, and they were characterized by using an optical microscope, Raman spectroscopy, scanning electron microscopy (SEM), and LCR meter. 

## 2. Materials and Methods

GO, dispersed with a concentration of 0.1 wt % (1 mg/mL) in DI water, is negatively charged due to the oxygen functional group attached to the GO. In contrast, an SF surface tends to be positively charged due to static electricity. The static electricity occurs due to an electrical charge imbalance on the SF surface, which has a high electrical resistance to the charged carrier. The magnitude of the static electric force on the SF surface varies depending on the tendency of the electrons to move from the SF to another material when the SF contacts and separates from that material (Table S1). 

**Figure 1:**
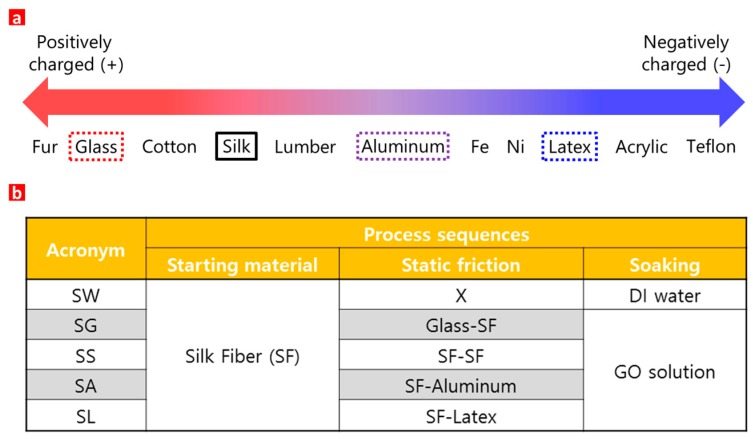
(**a**) Relative tendency of electrons to move from the SF when it contacts and separates from other materials [28]; (**b**) Various GO-coated SFs at various conditions.

**Figure 2:**
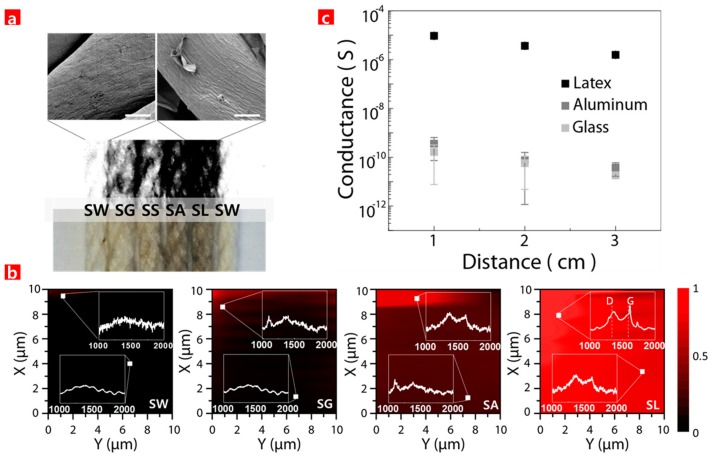
(**a**) Optical images of various GO-coated SFs, and SEM image of SW and SL (scale bar 10 μm); (**b**) Raman mapping image of SW, SG, SA, and SL, respectively; (**c**) Conductance of GO-coated SFs (SG, SA, and SL).

Figure 1a shows the relative tendency to lose (or gain) electrons on the surface, which eventually becomes positively (or negatively) charged when two different materials contact and separate. The material on the left tends to lose electrons and become positively charged, while that on the right tends to gain electrons and become negatively charged. Based on this phenomenon, the magnitude of the positive charge force on the SF can be controlled, which in turn enables the negatively charged GO coating on the SF to be controlled [37]. Various SFs with and without GO coatings at various conditions are shown in Figure 2. SG, SS, SA, and SL represent the SFs that were rubbed against a glass bar, nothing (control sample), aluminum foil, and latex gloves, respectively, for 3 min before submerging the processed SF in the GO solution (Figure S1); here the 3 min duration was considered sufficient to change the surface conditions. SW represents the SF that was submerged in the DI water instead of the GO solution (Figure S1). 

## 3. Results and Discussion

Figure 2a shows an optical image of various SFs (SW, SG, SS, SA, and SL) in sequential order. The SW is again located beside the SL in order to clearly distinguish the difference between the highly GO-coated SF (SL) and intact SF (SW). The SF images darkened as the GO coating on the SF thickened. This trend matched the expectations expressed in Figure 1a. The SEM images of the intact SF (SW) and highly GO-coated SF (SL) are also shown in the inset of Figure 2a and Figure S2. The chemical element analyses of the SL surface were conducted via energy-dispersive X-ray spectroscopy (EDS) (Figure S3). The results reveal that the GOs were seamlessly coated on the surface of the SF that was rubbed against latex before being soaked in the GO solution. 

The uniformity and quality of the GO coating on the SF were further investigated via a Raman analysis. No peaks were observed from the SW, but the peaks representing the GO began to be observed and were fully observed from the SA and SL, respectively, as shown in Figure 2b. The Raman mapping of the GO peaks was shown using colors in a linear scale with red and black representing the presence and absence of GO, respectively. For the integrated area from 1500 to 1650 cm^−1^ from the SL, the presence of GO was set at 1, and the absence of GO was set at 0. The Raman mapping results show that high quality GO was uniformly and seamlessly coated on the SL rubbed against the latex glove, while the GO was lightly coated on the SG rubbed against the glass bar. This clearly indicates that the surface of the SF rubbed against the latex became more positively charged than that rubbed against any other materials in this work. By extension, this SF surface attracted more GO. In addition, the Raman analysis clearly distinguished the SA and SL, as shown in Figure 2b, while the optical microscopic image in Figure 2a did not. The results from the Raman analysis also matched well with the SEM images, indicating the seamless GO coating on the SF surface. When the SF had a greater positive charge, the GO that has lots of partially negatively charged functional groups such as carboxyl and hydroxyl was better able to coat itself onto the SF surface, leading to a more conductive surface [16,26]. Several GO-coated SFs (SG, SA, and SL) were analyzed via conductance measurements. Figure 2c shows electrical conductance of the GO-coated SFs at various conditions as a function of distance. It shows that the GO-coated SF that was rubbed against latex had the highest conductivity, while the GO-coated SF that was rubbed against glass had the highest resistivity. Subsequently, this reveals that the SF rubbed against the latex was more positively charged, leading to a better GO coating on the SF. The conductance trend in Figure 2c, which was extracted from the from the transmission line method (TLM) in Figure S4, matched well with that of the optical image and Raman analysis in Figure 2a,b. From the above results, it was concluded that the SL surface was sufficiently coated with GO in the experimental results, as was expected given the conditions. Therefore, the SL was selected for further investigation. In the past, several GO coating methods on a desired substrate were developed using adhesive intermediates between the GO and the desired substrate in order to enhance adhesion. However, those intermediates often caused non-uniform GO coatings and were potential sources of impurities. In the present work, the application of the GO coating on the SF surface was carried out exclusively with electrical force. It should be noted that this method could be applied to any other substrate with a high resistance to a charged carrier. 

**Figure 3:**
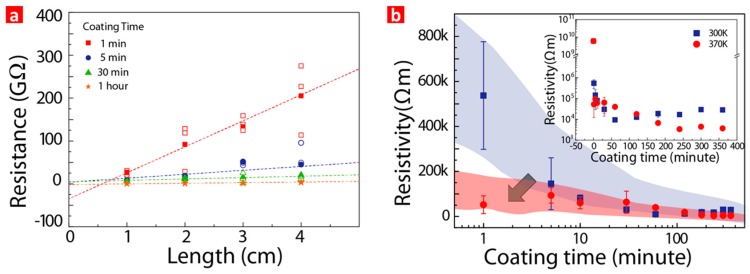
(**a**) SF resistance depending on distance at different coating times (Open symbols are experimental values whose average value is marked as solid symbol); (**b**) SF resistivity as a function of coating time at different coating temperatures.

The electrical properties of the GO-coated SF (SL) was further investigated at various coating times, as shown in Figure 3a, and at various GO solution temperatures, as shown in Figure 3b. Figure 3a shows the electrical resistance of the GO-coated SF (SL) rubbed against latex as a function of the distance at various GO coating times from the TLM patterns. This reveals that the sheet resistance of the SL decreased as the coating time increased. The sheet resistance of the SL was also quantitatively plotted as a function of the coating time at two different GO solution temperatures in Figure 3b. This reveals that the sheet resistance of the SL decreased in a log scale and eventually saturated after one hour coating time at 300 K. The saturation of the sheet resistance of the SL indicates that the process in which the negatively charged GO was coated onto the positively charged SF surface was self-limiting. This means that the attraction force of the SF toward the GO decreased as an increasing amount of GO was coated on the SF. Eventually, the attraction force became negligible because the positive charge on the SF was screened out by the attached GO. It was found that the thickness of the coated GO on the SF was around 70 nm at the saturated region. 

**Figure 4:**
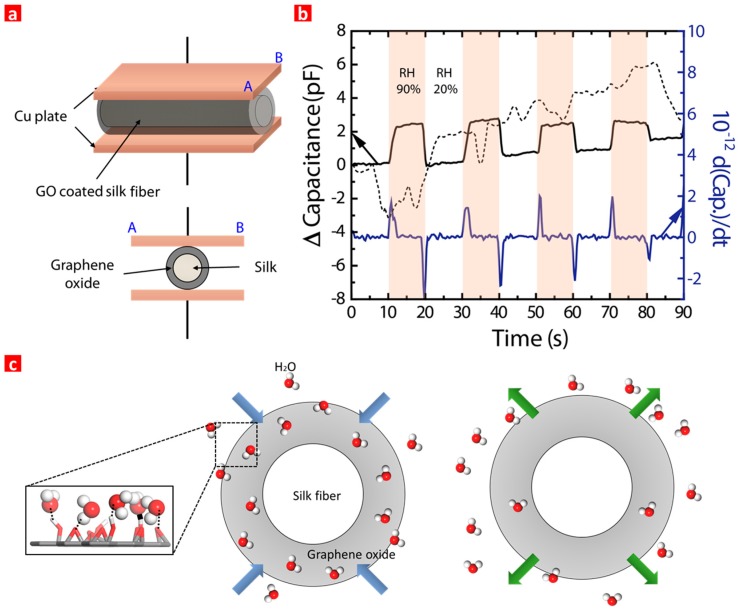
(**a**) Schematic image of capacitive humidity sensor where GO coated SF was implemented. The average diameter of SF is 0.17 cm; (**b**) The capacitance and its derivative curve of the sensor when the humidity changed between 20% RH and 90% RH. The dot represents the intact silk between the two cu plates; (**c**) Schematic image of H_2_O absorption (RH 90%) and desorption (RH 20%) characteristics on GO coated SF.

Finally, the SLs were implemented as an active sensing material between two Cu plates for capacitive humidity sensor applications, as shown in Figure 4a. The SL embedded humidity sensor was evaluated via the absorption-desorption dynamic cycles between 90% relative humidity (RH) and 20% RH at room temperature in Figure 4b. The sensor exhibited clear response-recovery behavior and well-behaved repeatability for humidity at room temperature. This superior sensing capability is attributed to the van der Waals forces between the hydroxyl and carboxyl bonds on the GO and H_2_O. The humidity in the air was quantified via the capacitance as shown in Figure 4c. For comparison, the SW without a GO coating was also investigated. The SW embedded sensor responded with water at first, but the value did not recover, indicating that the intact SF (SW) surface was soaked with water molecules and thus not usable for sensor applications. Furthermore, the derivative value of the capacitance in the absorption-desorption dynamic cycles was also monitored. It was found that this value provided clearer onset signals of water absorption and desorption. Generally, the processed derivative value of the output signal is preferred over the original output signal for image processing. Hence, clear response-recovery behavior and well-behaved repeatability for humidity was exhibited using the GO-coated SF as an active sensing material at room temperature. SF was charged with GO via electrical force, which renders it a potential candidate material and coating method for advanced humidity sensor applications. 

## 4. Conclusions

In summary, flexible silk fiber (SF) was coated with graphene oxide (GO) via electrostatic force without adhesive intermediates. It was found that the electrostatic force, i.e., the adhesion force, between the GO and SF could be controlled. The GO was uniformly and seamlessly coated on the SF in a large area, which was characterized by optical image, secondary electron microscopy (SEM), energy-dispersive X-ray spectroscopy (EDS), and a Raman analysis. In addition, the electrical properties of the GO-coated SF were investigated as a function of coating time and temperature. The GO-coated SF showed the feasibility of capacitive humidity sensor, unlike the intact SF. This approach allows the humidity sensor to take advantage of GO’s excellent sensing properties and flexibility. Therefore, it is expected that this method will enable the production of flexible, low power consumption devices at relatively low costs. 

## Supplementary Materials

The following are available online at www.mdpi.com/1424-8220/17/2/407/s1: Table S1. Triboelectric table predicting which will become positive vs. negative and how strong the effect will be. Figure S1. Schematic drawing of various GO coating process on SF. For comparison, DI-soaked SF (SW) was also prepared. Figure S2. (a) and (b) SEM images of GO-coated SF (SL) (scale bar: 1μm). Figure S3. EDS images of GO-coated SF (SL) along with the quantitative data. (a) SEM image of the SL, (b) the elemental maps using both C K and O K, (c) C K, and (d) O K, (scale bar : 20um). Figure S4. Resistance of GO-coated SF at various conditions as a function of distance; SFs were rubbed with each material (glass, aluminum, and latex) before soaking in GO solution.
