# Supplementary material for: Compliment Graphene Oxide Coating on Silk Fiber Surface via Electrostatic Force for Capacitive Humidity Sensor Applications
Source: Sensors (Basel). 2017 Feb 19;17(2):407. doi: 10.3390/s17020407 (PMC5335967; doi:10.3390/s17020407)
Supplement: Supplementary file 1 [file sensors-17-00407-s001.pdf]

Supporting Information

Compliment Graphene Oxide Coating on Silk Fiber Surface via Electrostatic Force for Capacitive Humidity Sensor Applications

Kook In Han, Seungdu Kim, In Gyu Lee, Jong Pil Kim, Jung-Ha Kim, Suck Won Hong, Byung Jin Cho, and Wan Sik Hwang

Table S1. Triboelectric table predicting which will become positive vs. negative and how strong the effect will be.[1]

Insulator name	Charge affinity nC/J	Metal effect[2]
Polyurethane foam	+60	+N
Sorbothane	+58	−W
Box sealing tape (BOPP)	+55	+W
Hair, oily skin	+45	+N
Solid polyurethane, filled	+40	+N
Magnesium fluoride (MgF_2)	+35	+N
Nylon, dry skin	+30	+N
Machine oil	+29	+N
Nylatron (nylon filled with MoS_2)	+28	+N
Glass (soda)	+25	+N
Paper (uncoated copy)	+10	−W
Wood (pine)	+7	−W
GE brand Silicone II (hardens in air)	+6	+N
Cotton	+5	+N
Nitrile rubber	+3	−W
Wool	0	−W
Polycarbonate	−5	−W
ABS	−5	−N
Acrylic (polymethyl methacrylate) and adhesive side of clear carton-sealing and office tape	−10	−N
Epoxy (circuit board)	−32	−N
Styrene-butadiene rubber (SBR, Buna S)	−35	−N
Solvent-based spray paints	−38	−N
PET (mylar) cloth	−40	−W
PET (mylar) solid	−40	+W
EVA rubber for gaskets, filled	−55	−N
Gum rubber	−60	−N
Hot melt glue	−62	−N
Polystyrene	−70	−N
Polyimide	−70	−N
Silicones (air harden & thermoset, but *not* GE)	−72	−N
Vinyl: flexible (clear tubing)	−75	−N
Carton-sealing tape (BOPP), sanded down	−85	−N
Olefins (alkenes): LDPE, HDPE, PP	−90	−N
Cellulose nitrate	−93	−N
Office tape backing (vinyl copolymer?)	−95	−N
UHMWPE	−95	−N
Neoprene (polychloroprene, *not* SBR)	−98	−N
PVC (rigid vinyl)	−100	−N
Latex (natural) rubber	−105	−N
Viton, filled	−117	−N

Epichlorohydrin rubber, filled	−118	−N
Santoprene rubber	−120	−N
Hypalon rubber, filled	−130	−N
Butyl rubber, filled	−135	−N
EDPM rubber, filled	−140	−N
Teflon	−190	−N

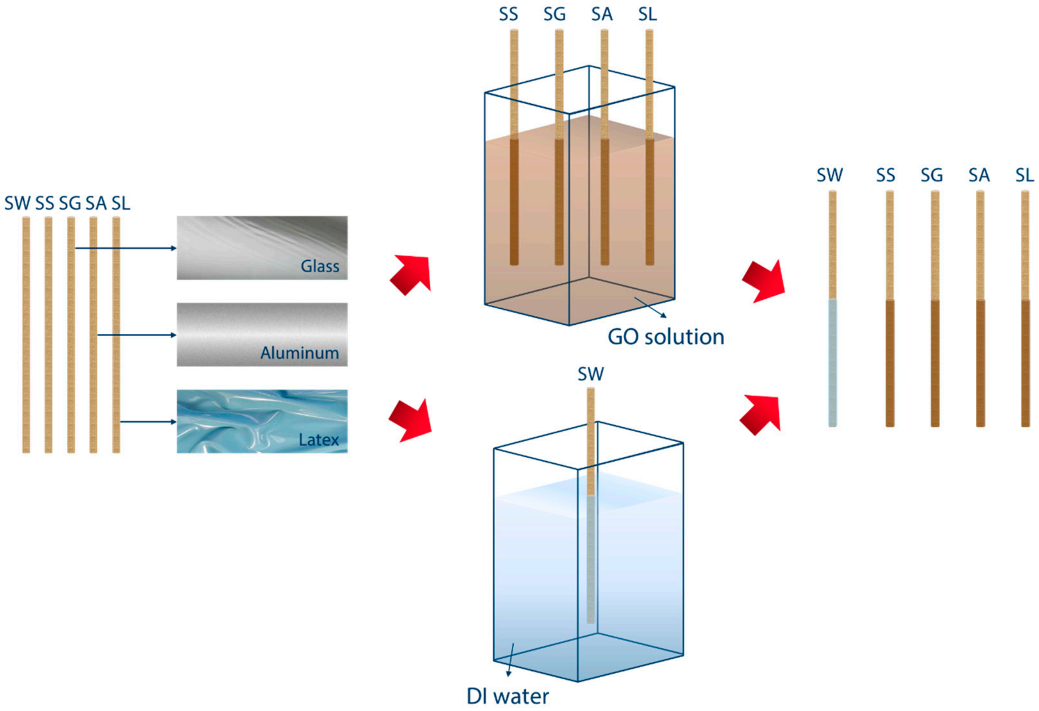

Figure S1. Schematic drawing of various GO coating process on SF. For comparison, DI-soaked SF (SW) was also prepared.

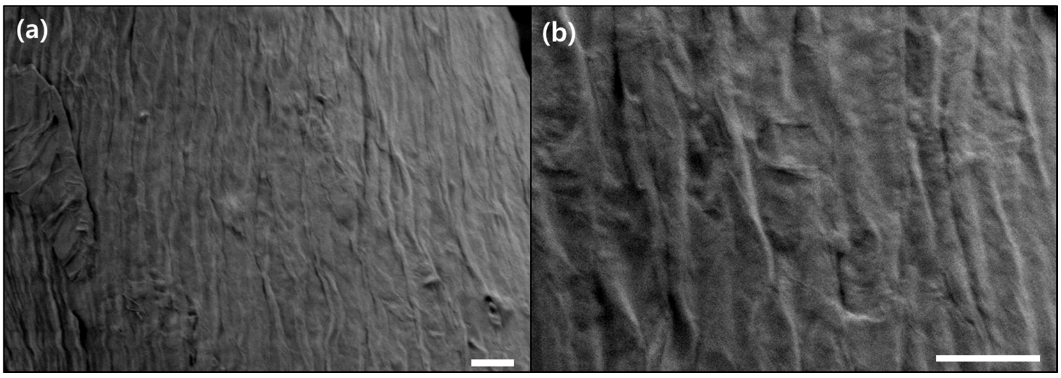

Figure S2. (**a**) and (**b**) SEM images of GO-coated SF (SL) (scale bar: 1μm)

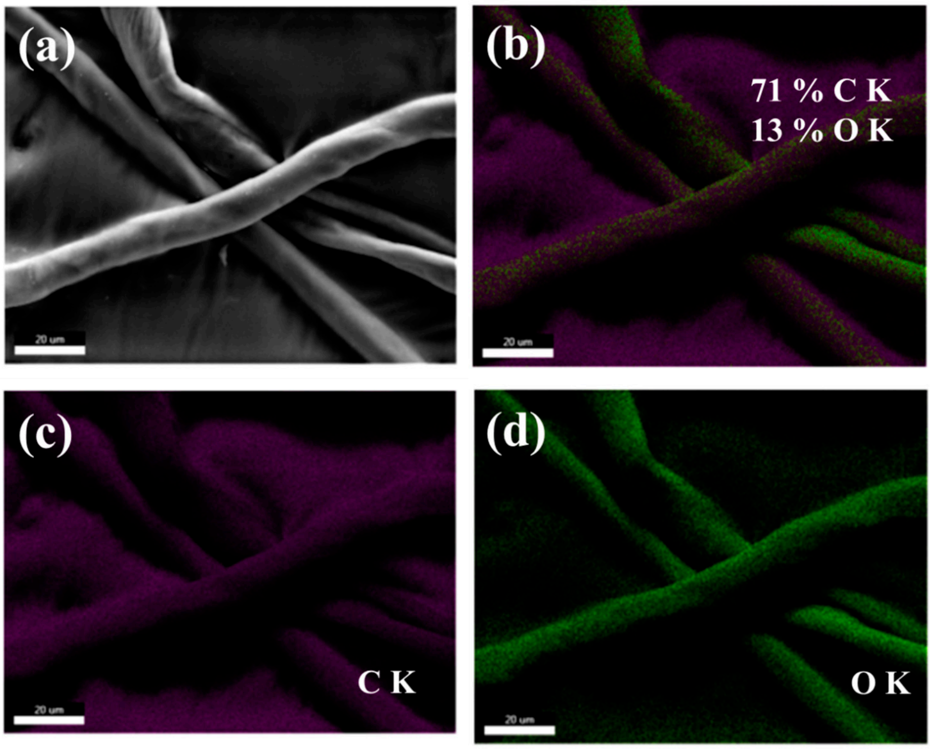

Figure S3. EDS images of GO-coated SF (SL) along with the quantitative data. (**a**) SEM image of the SL, (**b**) the elemental maps using both C K and O K, (**c**) C K, and (**d**) O K. (scale bar : 20um)

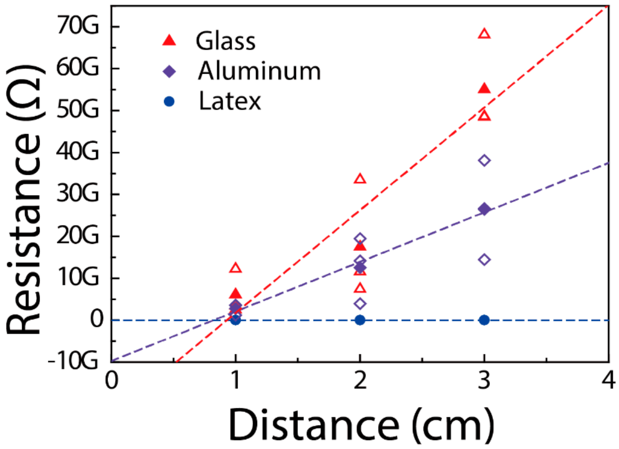

Figure S4. Resistance of GO-coated SF at various conditions as a function of distance; SFs were rubbed with each material (glass, aluminum, and latex) before soaking in GO solution.

Supplementary References

1 Tests were performed by Bill Lee (Ph.D., physics) ©2009 by AlphaLab, Inc. (TriField.com), which also manufactured the test equipment used. This table may be reproduced only if reproduced in whole.
2 Charge acquired if rubbed with metal (W = weak, N = normal, or consistent with the affinity).
